# Supplementary material for: Host Recognition and Specific Infection of Endomelanconiopsis endophytica during Early Infection
Source: J Fungi (Basel). 2023 Oct 23;9(10):1040. doi: 10.3390/jof9101040 (PMC10607883; doi:10.3390/jof9101040)
Supplement: Supplementary file 1 [file jof-09-01040-s001.zip › jof-2662021-supplementary.pdf]

Supplementary materials

Table S1. Prediction results of gene function in *Endomelanconiopsis endophytica*.

Databases	GO	KOG	CAZy	KEGG	NR	Pfam	TCDB
Endomelanconiopsis endophytica	5,747	2,123	465	7,836	8,088	5747	512

GO, Gene Ontology; COG, Cluster of Orthologous Groups of proteins; CAZy, Carbohydra-Active enZYmes Database; KEGG, Kyoto Encyclopedia of Genes and Genomes; NR, Non-Redundant Protein Database; Pfam, the Protein families database; TCDB, Transporter Classification Database.

Table S2. Quality control of transcriptomic data in *Endomelanconiopsis endophytica*.

Sample name	A_CK_1	A_CK_2	A_CK_3	B_2hpi_1	B_2hpi_2	B_2hpi_3	C_6hpi_1	C_6hpi_2	C_6hpi_3
Total Reads Count (#)	40266100	50124544	39480446	35727680	38976230	40435252	40327524	38415162	41369378
Total Bases Count (bp)	5.73E+09	7.18E+09	5.61E+09	5.1E+09	5.54E+09	5.79E+09	5.69E+09	5.45E+09	5.76E+09
Average Read Length (bp)	142.2	143.23	142.11	142.82	142.07	143.12	141.05	141.81	139.24
Q10 Bases Count (bp)	5.73E+09	7.18E+09	5.61E+09	5.1E+09	5.54E+09	5.79E+09	5.69E+09	5.45E+09	5.76E+09
Q10 Bases Ratio (%)	100.00%	100.00%	100.00%	100.00%	100.00%	100.00%	100.00%	100.00%	100.00%
Q20 Bases Count (bp)	5.63E+09	7.07E+09	5.52E+09	5E+09	5.45E+09	5.7E+09	5.59E+09	5.35E+09	5.66E+09
Q20 Bases Ratio (%)	98.34%	98.53%	98.31%	98.05%	98.50%	98.55%	98.21%	98.19%	98.34%
Q30 Bases Count (bp)	5.41E+09	6.82E+09	5.29E+09	4.78E+09	5.26E+09	5.5E+09	5.35E+09	5.12E+09	5.44E+09
Q30 Bases Ratio (%)	94.47%	94.98%	94.38%	93.64%	94.92%	95.05%	94.10%	94.05%	94.48%
N Bases Count (bp)	188029	161610	182234	136642	136523	129823	157384	167344	187691
N Bases Ratio (%)	0.00%	0.00%	0.00%	0.00%	0.00%	0.00%	0.00%	0.00%	0.00%
GC Bases Count (bp)	3.38E+09	4.24E+09	3.29E+09	2.95E+09	3.28E+09	3.44E+09	3.35E+09	3.23E+09	3.22E+09
GC Bases Ratio (%)	59.06%	59.00%	58.58%	57.76%	59.17%	59.48%	58.90%	59.37%	55.83%

Continued for Table S2. Quality control of *Endomelanconiopsis endophytica* transcriptomic data in temporal infection experiment.

Sample name	D_12hpi_1	D_12hpi_2	D_12hpi_3	E_24hpi_1	E_24hpi_2	E_24hpi_3	F_48hpi_1	F_48hpi_2	F_48hpi_3
Total Reads Count (#)	40255580	38201628	42191358	32995466	39290272	39218962	37053004	40829590	43408170
Total Bases Count (bp)	5.56E+09	5.23E+09	6E+09	4.52E+09	5.32E+09	5.38E+09	5.04E+09	5.77E+09	6.11E+09
Average Read Length (bp)	138.2	136.88	142.19	136.89	135.31	137.17	136.06	141.24	140.8
Q10 Bases Count (bp)	5.56E+09	5.23E+09	6E+09	4.52E+09	5.32E+09	5.38E+09	5.04E+09	5.77E+09	6.11E+09
Q10 Bases Ratio (%)	100.00%	100.00%	100.00%	100.00%	100.00%	100.00%	100.00%	100.00%	100.00%
Q20 Bases Count (bp)	5.47E+09	5.14E+09	5.9E+09	4.44E+09	5.23E+09	5.28E+09	4.95E+09	5.66E+09	6.01E+09
Q20 Bases Ratio (%)	98.38%	98.23%	98.29%	98.27%	98.29%	98.20%	98.18%	98.08%	98.29%
Q30 Bases Count (bp)	5.26E+09	4.92E+09	5.66E+09	4.26E+09	5.02E+09	5.06E+09	4.74E+09	5.41E+09	5.77E+09
Q30 Bases Ratio (%)	94.61%	94.18%	94.33%	94.30%	94.41%	94.13%	94.10%	93.74%	94.35%
N Bases Count (bp)	180339	157472	182132	149243	175244	162145	150537	161818	182053
N Bases Ratio (%)	0.00%	0.00%	0.00%	0.00%	0.00%	0.00%	0.00%	0.00%	0.00%
GC Bases Count (bp)	3.31E+09	3.05E+09	3.5E+09	2.66E+09	3.12E+09	3.2E+09	2.95E+09	3.32E+09	3.49E+09
GC Bases Ratio (%)	59.46%	58.35%	58.39%	58.83%	58.74%	59.43%	58.58%	57.59%	57.08%

Table S2. Quality control of transcriptomic data in *Endomelanconiopsis endophytica*.

Sample name	A_CK_1	A_CK_2	A_CK_3	B_2hpi_1	B_2hpi_2	B_2hpi_3	C_6hpi_1	C_6hpi_2	C_6hpi_3
Total Reads Count (#)	40266100	50124544	39480446	35727680	38976230	40435252	40327524	38415162	41369378
Total Bases Count (bp)	5.73E+09	7.18E+09	5.61E+09	5.1E+09	5.54E+09	5.79E+09	5.69E+09	5.45E+09	5.76E+09
Average Read Length (bp)	142.2	143.23	142.11	142.82	142.07	143.12	141.05	141.81	139.24
Q10 Bases Count (bp)	5.73E+09	7.18E+09	5.61E+09	5.1E+09	5.54E+09	5.79E+09	5.69E+09	5.45E+09	5.76E+09
Q10 Bases Ratio (%)	100.00%	100.00%	100.00%	100.00%	100.00%	100.00%	100.00%	100.00%	100.00%
Q20 Bases Count (bp)	5.63E+09	7.07E+09	5.52E+09	5E+09	5.45E+09	5.7E+09	5.59E+09	5.35E+09	5.66E+09
Q20 Bases Ratio (%)	98.34%	98.53%	98.31%	98.05%	98.50%	98.55%	98.21%	98.19%	98.34%
Q30 Bases Count (bp)	5.41E+09	6.82E+09	5.29E+09	4.78E+09	5.26E+09	5.5E+09	5.35E+09	5.12E+09	5.44E+09
Q30 Bases Ratio (%)	94.47%	94.98%	94.38%	93.64%	94.92%	95.05%	94.10%	94.05%	94.48%
N Bases Count (bp)	188029	161610	182234	136642	136523	129823	157384	167344	187691
N Bases Ratio (%)	0.00%	0.00%	0.00%	0.00%	0.00%	0.00%	0.00%	0.00%	0.00%
GC Bases Count (bp)	3.38E+09	4.24E+09	3.29E+09	2.95E+09	3.28E+09	3.44E+09	3.35E+09	3.23E+09	3.22E+09
GC Bases Ratio (%)	59.06%	59.00%	58.58%	57.76%	59.17%	59.48%	58.90%	59.37%	55.83%

Table S3. Gene annotations of top 10 expressed effectors in *Endomelanconiopsis endophytica*.

Gene ID	Effector type	DFVF	PHI-base	CAZy	GO	KOG	NR	Pfam	KEGG
A0194	Apoplastic	-	-	-	-	-	putative -binding protein	-	-
A0439	Apoplastic	Q8J0U4_LEPMC	PHI:2532	-	-	-	putative epl1 protein	-	-
A1948	Apoplastic	-	-	GH13_1	GO:0003824	G	putative alpha-amylase a type-1/2 protein	PF09260	[PATH: ko00500] [PATH: ko01100] [PATH: ko04973]
A3211	Apoplastic	-	-	-	GO:0009277	-	hypothetical protein	PF01185	-
A4087	Apoplastic	-	-	-	-	-	hypothetical protein	-	-
A4417	Apoplastic	-	-	-	-	-	hypothetical protein	-	-
A5728	Apoplastic	-	-	-	-	-	hypothetical protein	-	-
A6540	Apoplastic	-	-	-	-	-	putative candidate effector 5 protein	-	-
A8041	Apoplastic	-	-	-	-	-	hypothetical protein	-	-
A8732	Apoplastic	-	-	-	-	-	putative gpi anchored cell wall protein	-	-

DFVF, Database of Fungal Virulence Factors; PHI-base, Pathogen-Host Interactions database; GO, Gene Ontology; COG, Cluster of Orthologous Groups of proteins; CAZy, Carbohydra-Active enZYmes Database; KEGG, Kyoto Encyclopedia of Genes and Genomes; NR, Non-Redundant Protein Database; Pfam, the Protein families database; TCDB, Transporter Classification Database

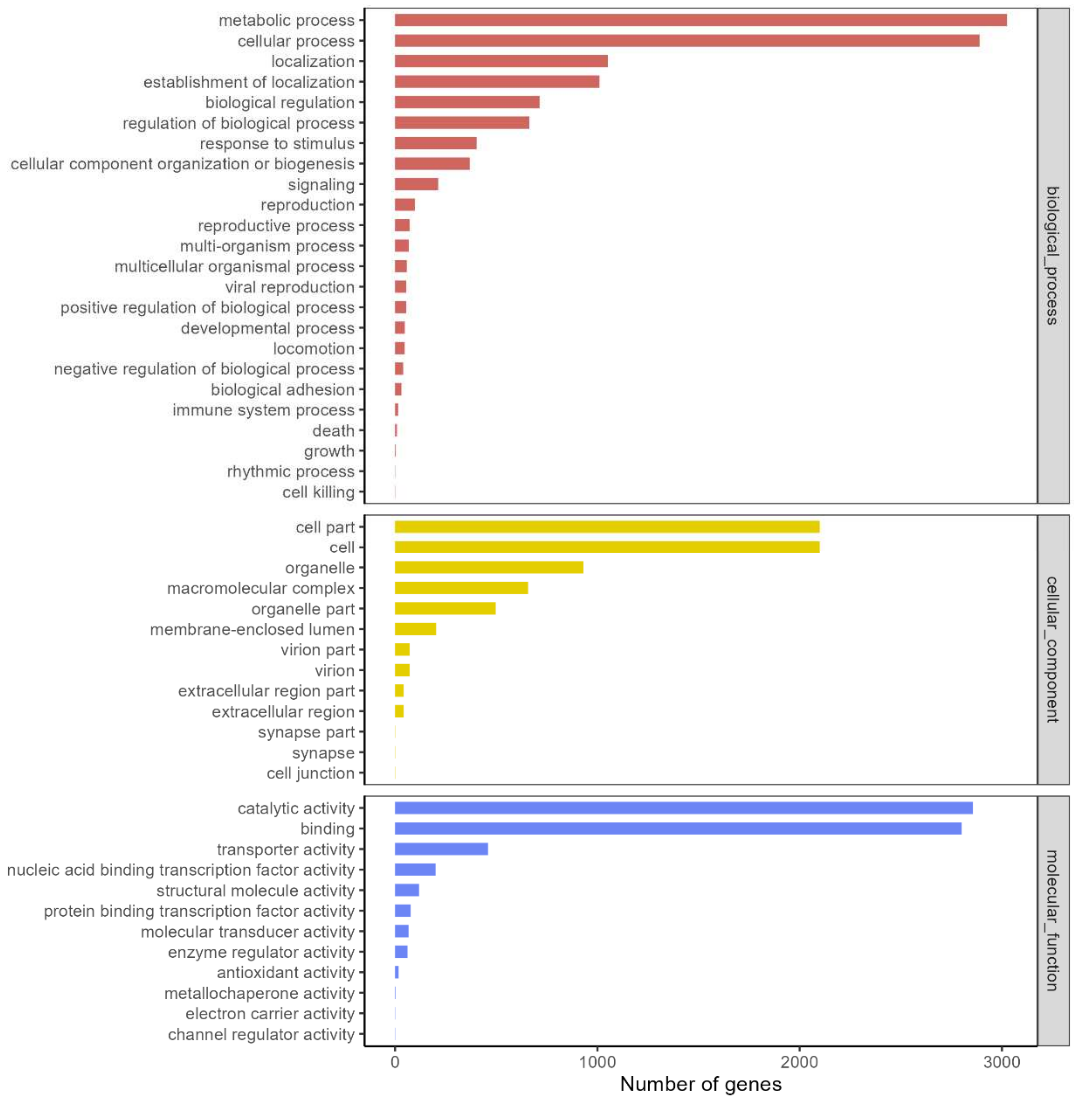

Figure S1. GO annotation of *Endomelanconiopsis endophytica*. The abscissa represents the number of genes, while the ordinate represents the names of the GO classes. The color represents the GO ontology.

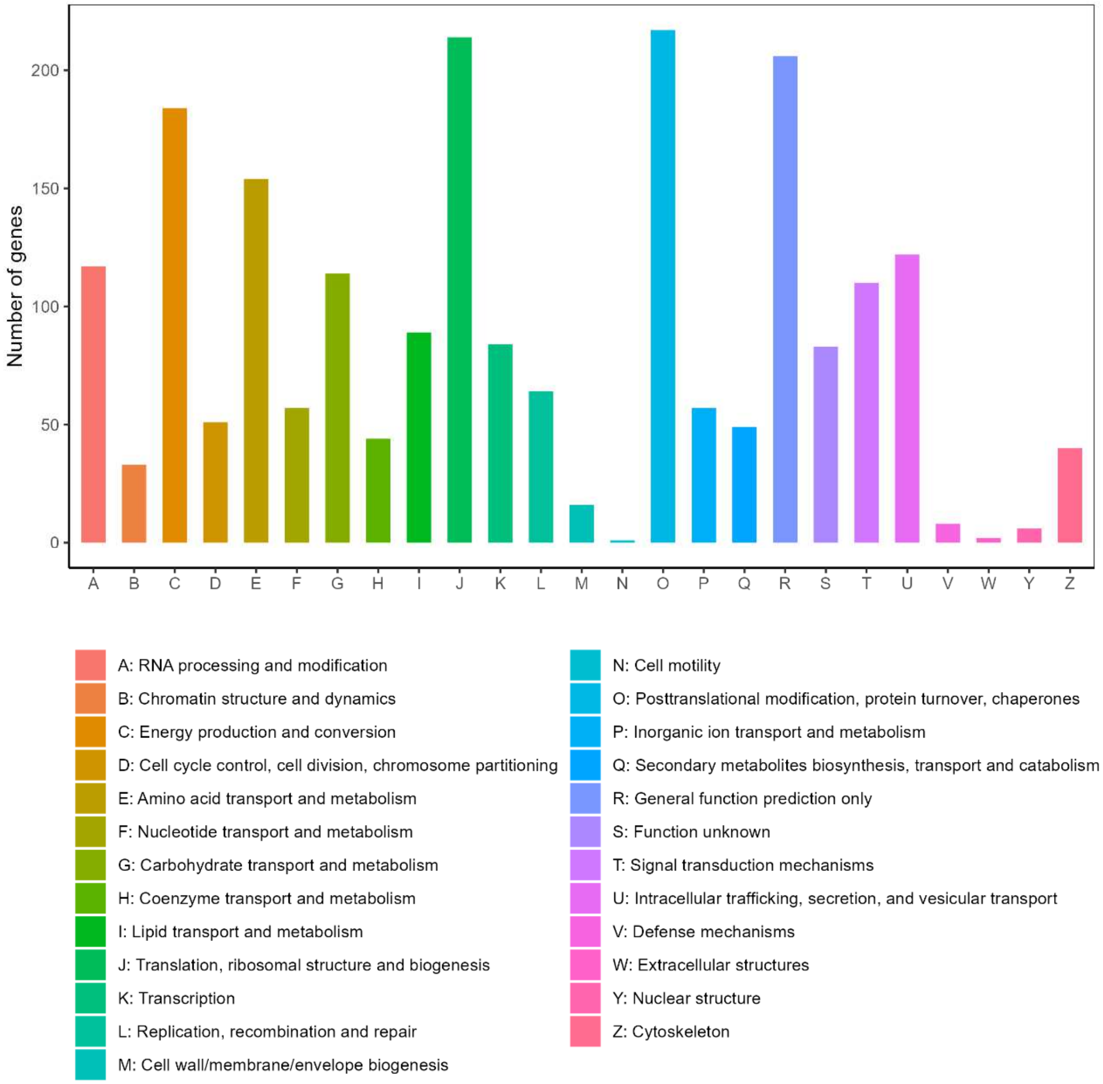

Figure S2. KOG annotation of *Endomelanconiopsis endophytica*. The abscissa represents the abbreviations of the KOG classes, while the ordinate represents the number of genes. The legend represents the names of KOG classes.

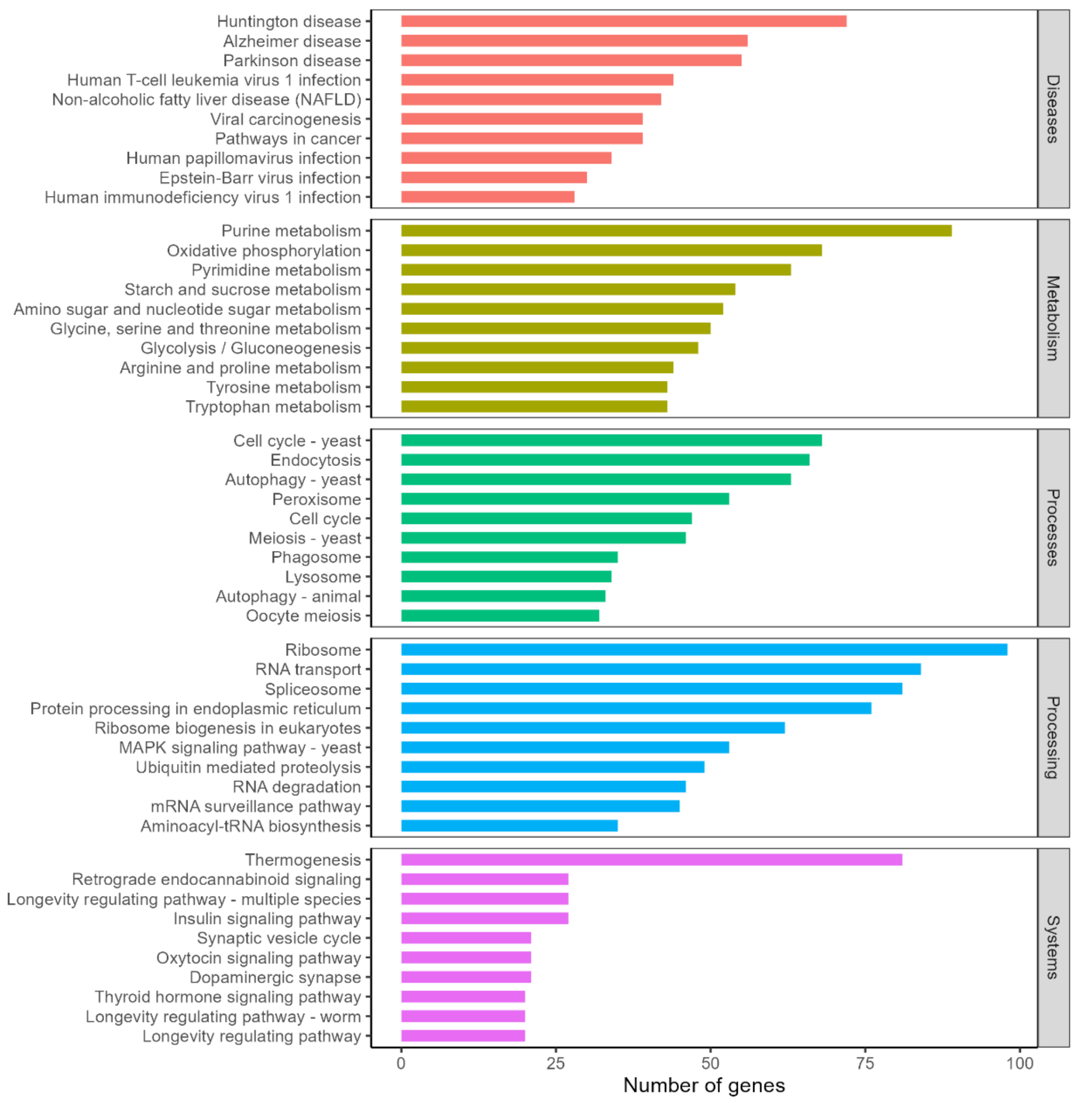

Figure S3. Top 10 pathway in each Kyoto Encyclopedia of Genes and Genomes (KEGG) class annotation of *Endomelanconiopsis endophytica*. The abscissa represented the number of genes, while the ordinate represented the names of KEGG pathway. The color represented the KEGG classes.

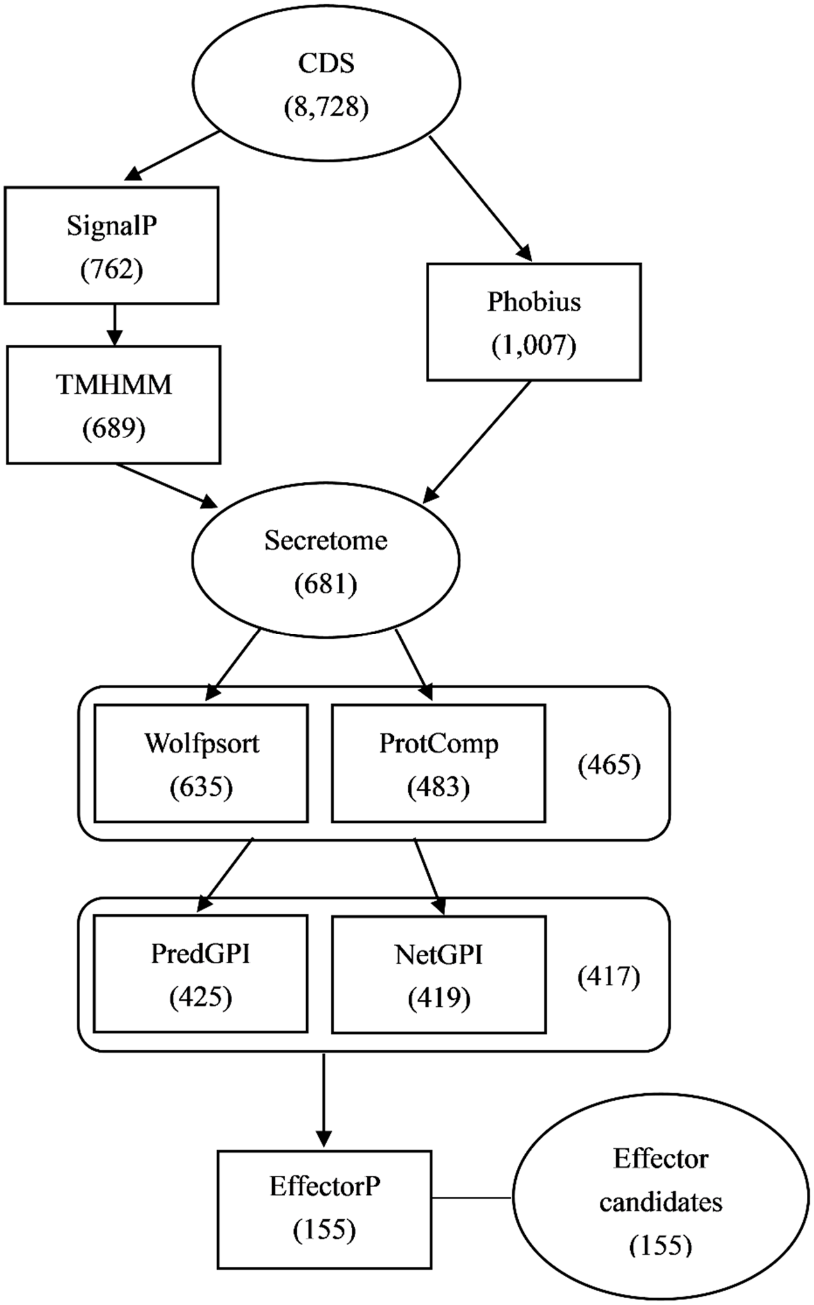

Figure S4. Workflow demonstrating the prediction process of effector candidates in *Endomelanconiopsis endophytica*.

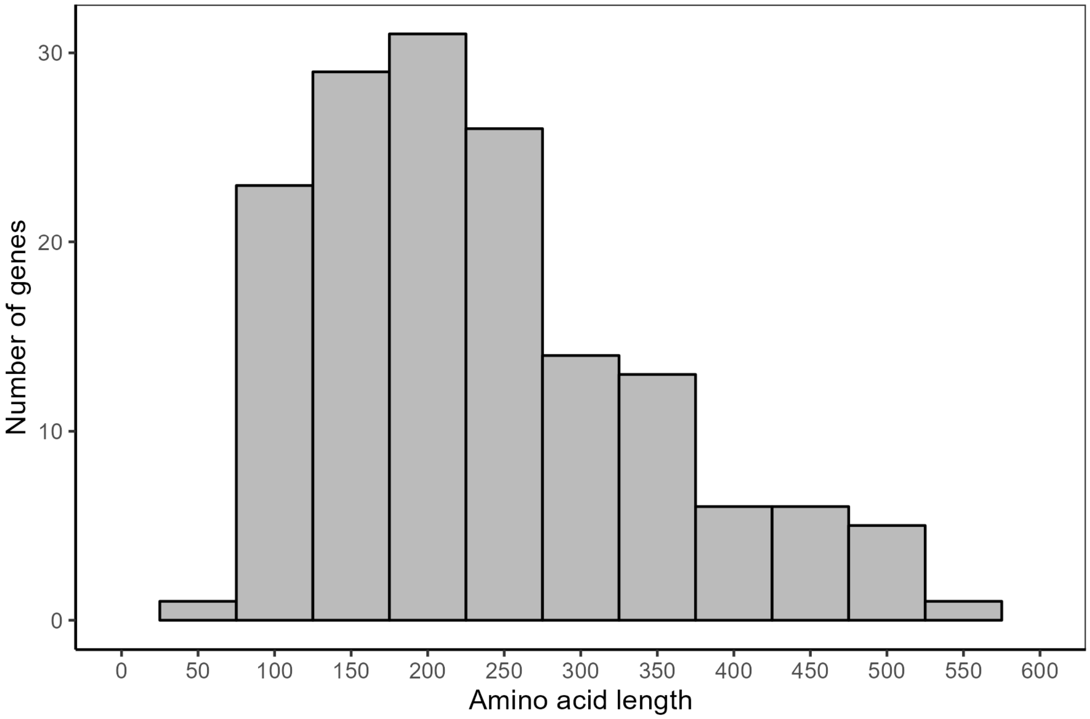

Figure S5. Amino acid length frequency distribution of effector candidates in *Endomelanconiopsis endophytica*. The abscissa represented amino acid length, while the ordinate represented the counts of genes.

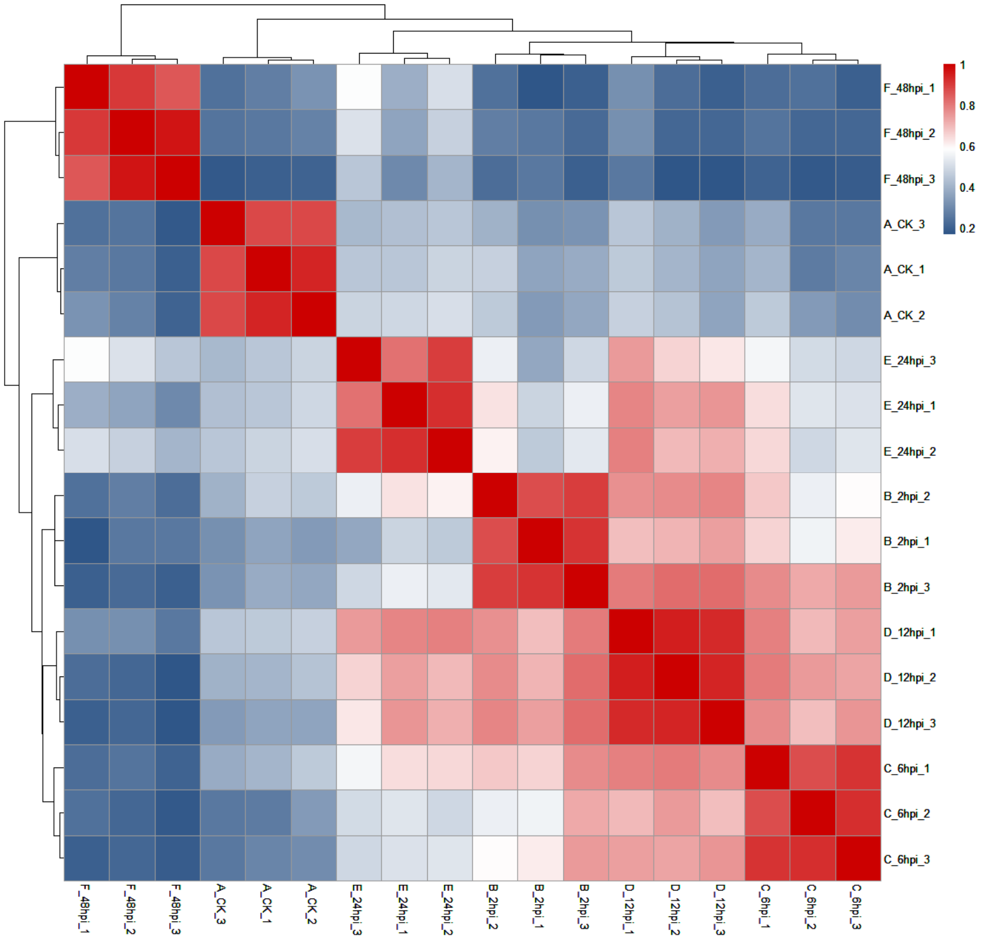

Figure S6. Correlation heatmap of transcriptome samples during the infection process of *Castanopsis fissa* by *Endomelanconiopsis endophytica*. The hierarchical clustering among samples is shown on the left and top. The correlation is reflected by the color change, and red to blue indicates the Pearson's correlation coefficient values from high to low.

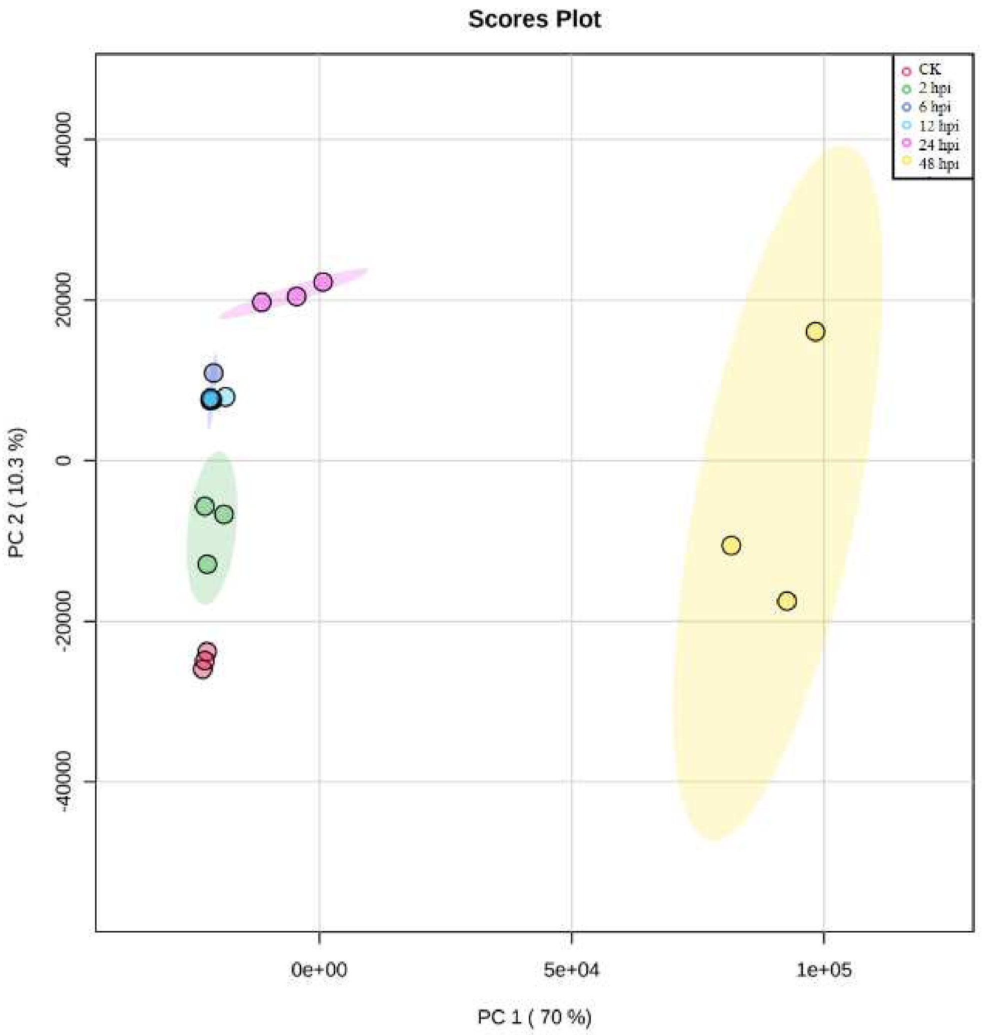

Figure S7. Principal component analysis of the transcriptome samples during the infection process of *Castanopsis fissa* by *Endomelanconiopsis endophytica*. The abscissa represents component 1, while the ordinate represents component 2. Different colors represent groups at different times post infection. Dots in the same color represent repeated samples within a group, and ellipses are 95% confidence ellipses for samples in the same group.

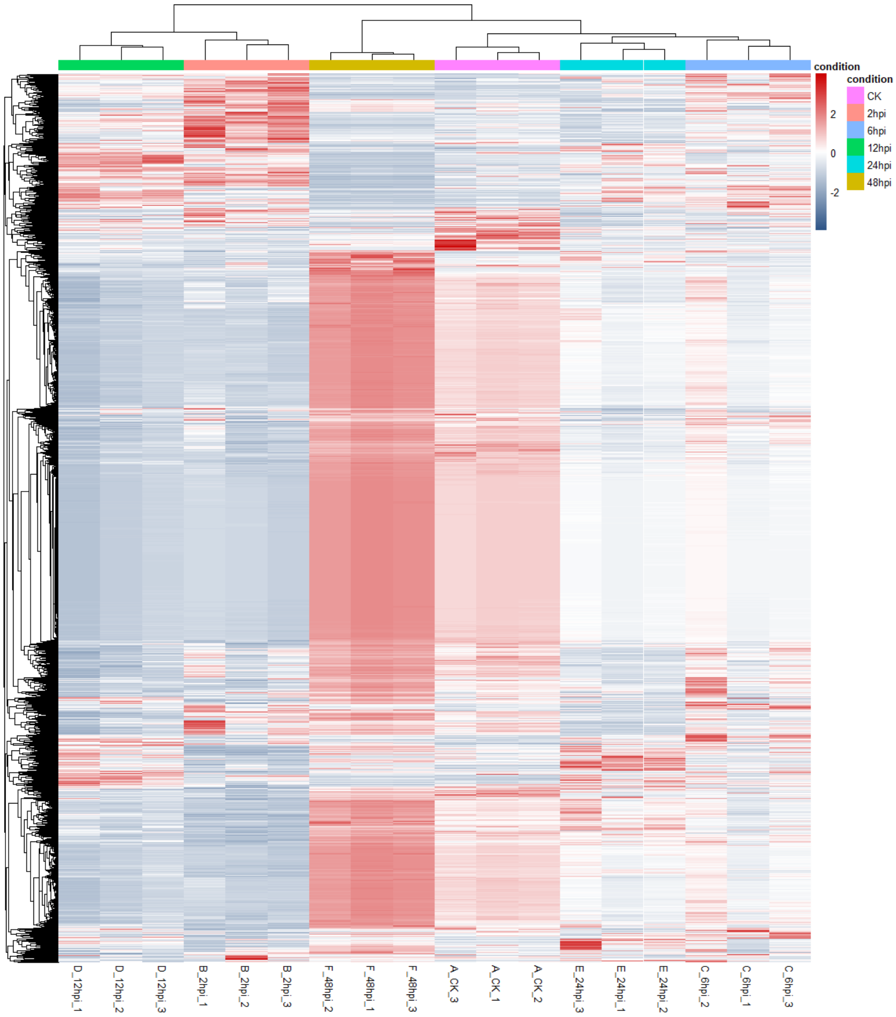

Figure S8. Differential gene cluster heat map of gene expressions in the infection process of *Castanopsis fissa* by *Endomelanconiopsis endophytica*. The abscissa represented the sample name and hierarchical clustering among samples, and the ordinate represented the differential genes and hierarchical clustering among genes.

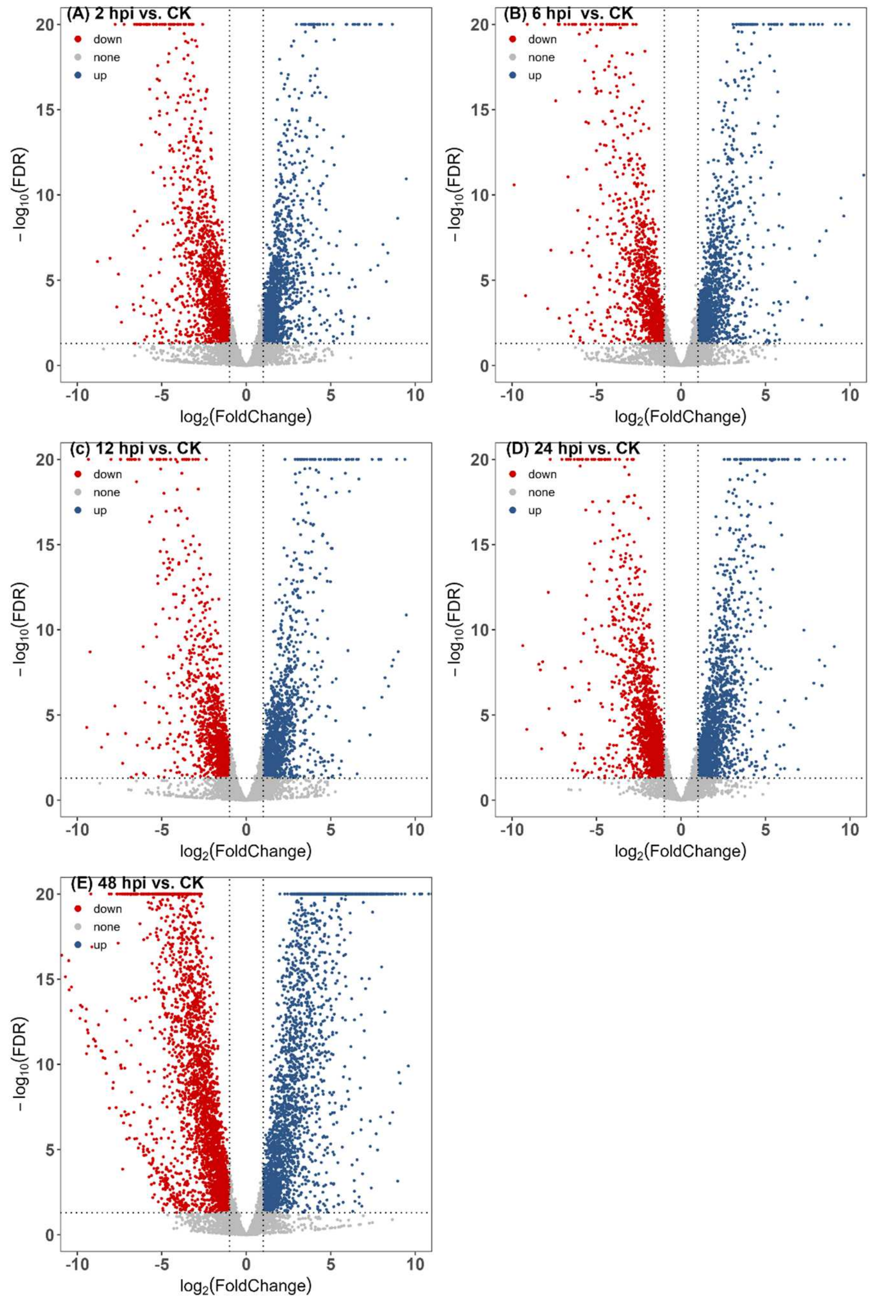

Figure S9. Differential gene volcano map of gene expression during the infection process of *Castanopsis fissa* by *Endomelanconiopsis endophytica*. (A) 2 hpi *vs.* CK, (B) 6 hpi *vs.* CK, (C) 12 hpi *vs.* CK, (D) 24 hpi *vs.* CK, (E) 48 hpi *vs.* CK. The results are based on DESeq2 analysis, and genes with | $\log_2$(Fold Change) | > 1 and q-value < 0.05 are considered as differentially expressed. Red points represent downregulated genes, blue points represent upregulated genes, and gray points represent no significant gene expression regulation.

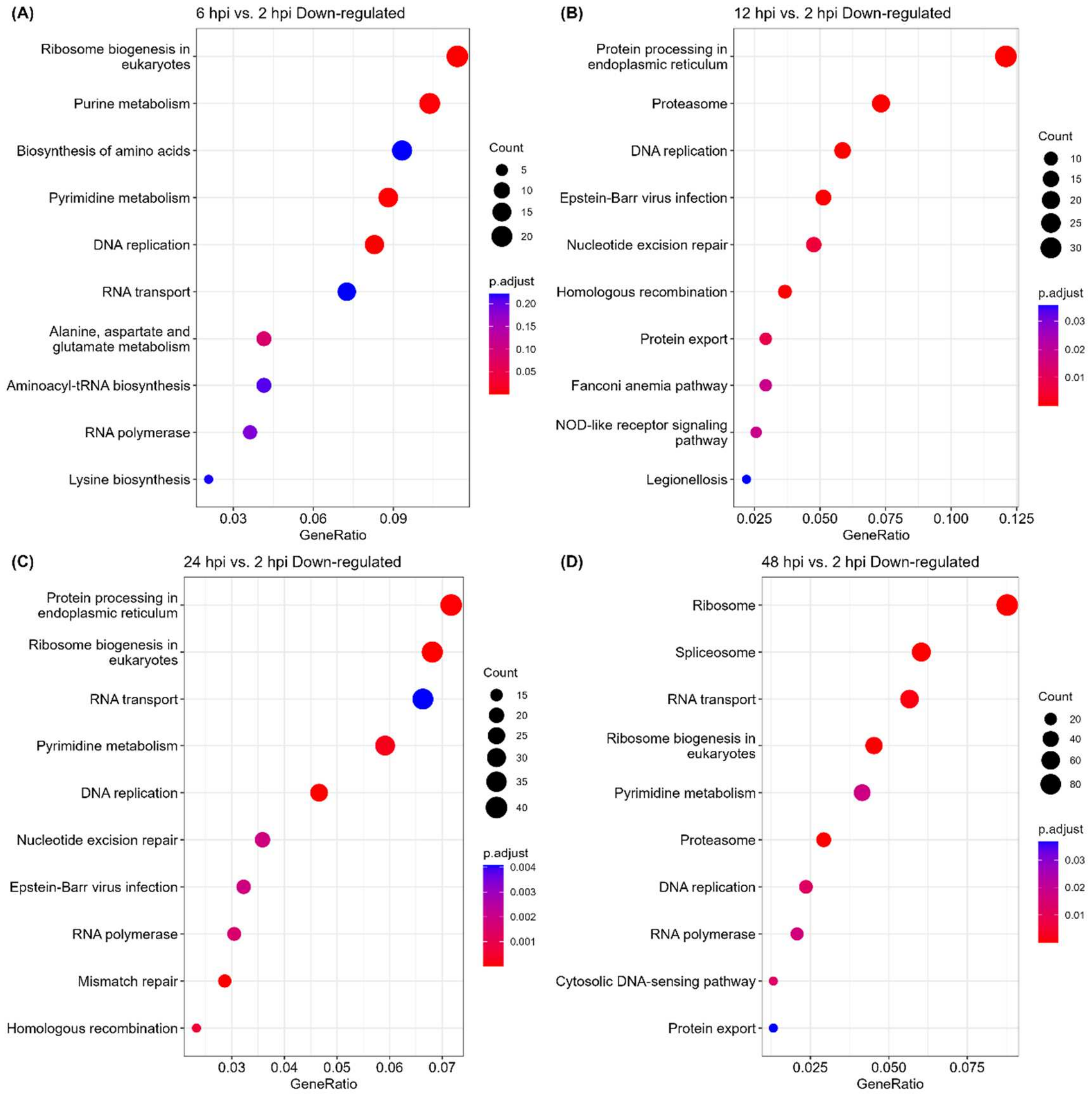

Figure S10. Pathway enrichment analysis of the downregulated genes in the infection process of *Castanopsis fissa* by *Endomelanconiopsis endophytica*. (A) 6 hpi *vs.* 2 hpi, (B) 12 hpi *vs.* 2 hpi, (C) 24 hpi *vs.* 2 hpi, (D) 48 hpi *vs.* 2 hpi. The top 10 Kyoto Encyclopedia of Genes and Genomes (KEGG) downregulated pathways are shown, with the abscissa representing the gene ratio, and the ordinate represents the names of the KEGG pathways. The depth of the color represents the adjusted *P*-value. The size of the circle on the graph represents the number of genes.
